# Nonarteritic Anterior Ischemic Optic Neuropathy: Cystic Change in the Inner Nuclear Layer Caused by Edema and Retrograde Maculopathy

**DOI:** 10.1016/j.xops.2022.100230

**Published:** 2022-10-04

**Authors:** Anne-Catherine Chapelle, Jean-Marie Rakic, Gordon T. Plant

**Affiliations:** 1 Department of Ophthalmology, Central University Hospital of Liège, University of Liège, Liège, Belgium; 2 Department of Neurodegeneration and Rehabilitation, UCL Institute of Neurology, Faculty of Brain Sciences, University College London, London, United Kingdom

**Keywords:** Ischemic optic neuropathy, Microcystic macular edema, Neuro-ophthalmology, Retrograde maculopathy, Swollen disc, BCVA, best-corrected visual acuity, GCC, ganglion cell complex, INL, inner nuclear layer, LDL, low-density lipoprotein, MME, microcystic macular edema, NA-AION, nonarteritic anterior ischemic optic neuropathy, pRNFL, peripapillary retinal nerve fiber layer, RM, retrograde maculopathy, VF, automated perimetry

## Abstract

**Purpose:**

Microcystic macular edema (MME), also known as retrograde maculopathy (RM), is associated with severe optic atrophy because of a range of causes. However, similar changes have also been described in primary *retinal* pathology and the pathogenesis of MME is debated.

**Design:**

A retrospective observational case series.

**Participants:**

Patients with nonarteritic ischemic optic neuropathy.

**Methods:**

A retrospective observational case series was performed at the University Hospital of Liège, Belgium. The medical records of patients who were referred to our Neuro-ophthalmology department with a diagnosis of nonarteritic anterior ischemic optic neuropathy (NA-AION), between 2014 and 2021, were reviewed.

**Main Outcome Measures:**

Ganglion cell complex thickness, acute and chronic inner nuclear change.

**Results:**

In a cohort of 34 patients (mean age: 60 ± 12.5 years; 65.6% men) with NA-AION, we identified a transient microcystic change in the inner nuclear layer (INL) associated with optic disc swelling in 19 eyes at presentation. This early change was associated with a transudate of intraretinal and subretinal fluid originating from the optic disc. Among patients who had shown this transient change 3 subsequently developed MME, which remained fixed during the period of observation (range, 12–34 months). No MME was observed in patients without an early INL transient change. Microcystic macular edema was observed in patients with severe ganglion cell complex thinning at 6 months: mean (± SD) loss in superior hemimacula (−28.2 ± 5.2 μm [−33.3%, range, −22.3 to −30.3 μm]) and in inferior hemimacula (−30.7 ± 5.6 μm [−31.0%, range, −24.3 to 34.8 μm]).

**Conclusions:**

Our study has revealed 2 causes of INL cystic change in the same patients experiencing NA-AION, 1 reversible and the other likely permanent. This finding highlights the distinction between genuine edema related to transudation of fluid (in this case secondary to ischemic optic disc swelling) and the phenomenon observed in RM that is related to the degree of retinal nerve fiber layer/ganglion cell complex thinning. Cystic change in the INL is associated with severe optic atrophy (MME). However, similar changes have been described in *retinal* pathology and the pathogenesis of MME is debated.

Microcystic macular edema (MME) was first described in the context of OCT findings in multiple sclerosis by Gelfand et al.^1^ The abnormality was more likely to be seen in patients who had had a previous episode of optic neuritis and in cases with more marked thinning of the retinal nerve fiber and ganglion cell layers. Inner nuclear layer (INL) cell loss and thinning had previously been reported in a histopathologic study of the retina in multiple sclerosis, which also correlated with the severity of axonal loss, without mention of the presence of cystic change.^2^ The microcysts are located in the INL and show a perifoveal distribution.^1^^,^^3^ Microcystic macular edema is associated with severe optic atrophy found in the following: optic neuritis^1^^,^^4^; neuromyelitis optica^5^^,^^6^; hereditary optic neuropathies^7^^,^^8^; toxic and nutritional deficiency neuropathy^9^^,^^10^; glaucomatous neuropathy^11^^,^^12^; compressive optic neuropathy^13^; and infiltrative optic neuropathy, such as glioma.^14^^,^^15^ Abegg^14^ has suggested the term *retrograde maculopathy* (RM) for INL cystic change in this context.^16^ However, similar changes have also been described in *retinal* pathology, for example in the following: epiretinal membrane,^17^ age-related macular degeneration, diabetic retinopathy, and central serous retinopathy.^18^ Indeed, the earliest use of the term MME known to the authors (although in Romanian as *edemul macular microchistic*) was published in a description of an *isolated case* of suspected microcystic macular dystrophy in 1988. ^19^

We consider the following case series concerning 3 patients with nonarteritic anterior ischemic optic neuropathy (NA-AION) to be of particular interest in this context. These patients showed early acute *transient* microcystic changes in the INL associated with disc swelling in parallel with other evidence of retinal edema. Longstanding, perhaps permanent, MME was developed subsequently. The pathophysiologic basis for these 2 manifestations of INL cystic change following NA-AION will be discussed.

## Methods

A retrospective observational case series was performed at the University Hospital of Liège, Belgium. Twenty-three patients who experienced sudden painless monocular loss of vision were referred to our neuro-ophthalmology department or to the emergency department (9 cases). Recruitment took place between 2014 and 2021. The diagnosis of NA-AION was based on the anamnesis (painless sudden loss of vision involving 1 eye) and fundus examination. The latter showed a swollen disc in the affected eye and, in the fellow eye, either a crowded disc (typical of the *disc at risk* associated with NA-AION^20^, ^21^, ^22^) or sectoral atrophy indicating a previous episode of NA-AION. Spontaneous resolution of the optic disc swelling was noted within 8 weeks and the outcome was sectoral infarction. Giant cell arteritis was excluded by appropriate investigations. The clinical features were not compatible with a diagnosis of optic neuritis. Two patients were excluded because optic nerve head drusen were identified on OCT. Patients were White European, representing the typical local population of the clinic, except 1 who was Turkish. The mean age of the subjects was 60 ± 12.5 years (range, 22–88 years, median: 60.5) and 65.6% were men. A favorable opinion from the ethics committee of the University of Liège for the study had been received. The study adhered to the tenets of the declaration of Helsinki and a written informed consent was obtained for the 3 patients described in this case series.

The patients were all followed up at baseline and at 1, 3, and 6 months postepisode. Fundus fluorescein angiography and a macular spectral-domain OCT (Heidelberg Engineering) were performed in 27 patients at baseline: 6-line radial scans and 25-line raster scans centered on the fovea were obtained. Subsequently, patients were seen annually. Clinical examination included best-corrected visual acuity (BCVA, Snellen scale), slit-lamp examination, and fundus biomicroscopy. At follow-up, patients underwent automated perimetry (VF, Humphrey 24-2), with appropriate refractive correction and OCT (high-definition OCT Cirrus, Carl Zeiss Meditec), for which manual correction of segmentation was not possible. The thickness of the peripapillary retinal nerve fiber layer (pRNFL) and of the ganglion cell complex (GCC) (inner plexiform layer + ganglion cell layer) were evaluated at each visit along with en face OCT B-scan. Studies demonstrating poor image quality, not allowing adequate visualization of all retinal layers, were excluded. Scans were analyzed for the presence of intraretinal fluid and subretinal fluid by 3 skilled observers who were masked as to the aim of the study. For the 3 patients described in this series, INL thickness was also evaluated initially and at the latest follow-up visit. For INL thickness assessment, adequate segmentation was assessed and adjusted manually in 1 case with macular spectral domain OCT.

Grading of the acute optic disc swelling was conducted as a function of the mean RNFL thickness and the numbers of quadrant sectors involved (temporal, superior, inferior, nasal). For example, optic disc swelling showing pRNFL thickening in at least 1 sector to be < 120 μm was considered moderate. In the 3 cases described below, the swelling of the disc was considered severe because all sectors were involved (pRNFL thickness of all sectors > 120 μm) and the mean pRNFL thickness was high (mean [± SD], 257.7 ± 81 μm; range, 189–347 μm).

## Results

### Summary of Findings

**Figure 1:**
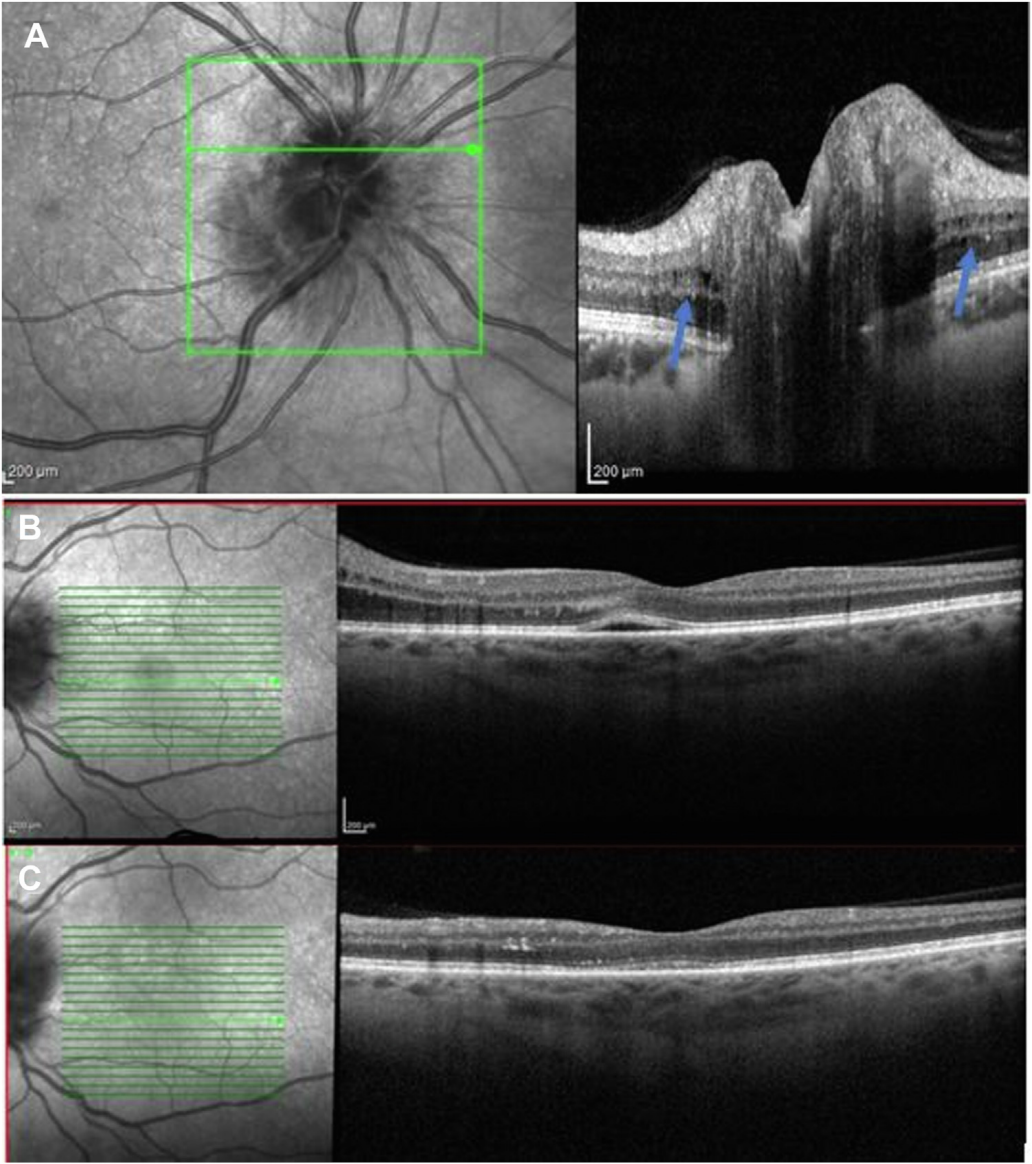
Spectral-domain OCT (Heidelberg Engineering) imaging. **A**, Microcystic change visible in the peripapillary region in moderate swelling of the disc (cohort case; blue arrows). **B**, Transudation of fluid through the retina at presentation. **C**, Twenty-three days later in 1 case selected from the 19 out 34 cases showing disc change without subsequent retrograde maculopathy (cohort case).

In a cohort of 34 patients (45 affected eyes and 23 healthy fellow eyes), we identified a transient microcystic change in the INL associated with optic disc swelling in 19 eyes at presentation. In these cases, the vacuoles were localized to the INL and were associated with transudation of intraretinal and subretinal fluid originating from the optic disc (see Fig 1 for 1 example). The microcystic change in these cases was transient and had resolved in all cases at 1 month (Fig 1). In cases with moderate optic disc swelling assessed in the global cohort, cystic changes were visible only in the peripapillary region (e.g., Fig 1). Four patients, who had shown this transient change subsequently developed MME that remained fixed during the period of observation (range, 12–34 months). The other cases (n = 30) did not show MME during the follow-up period.

**Figure 2:**
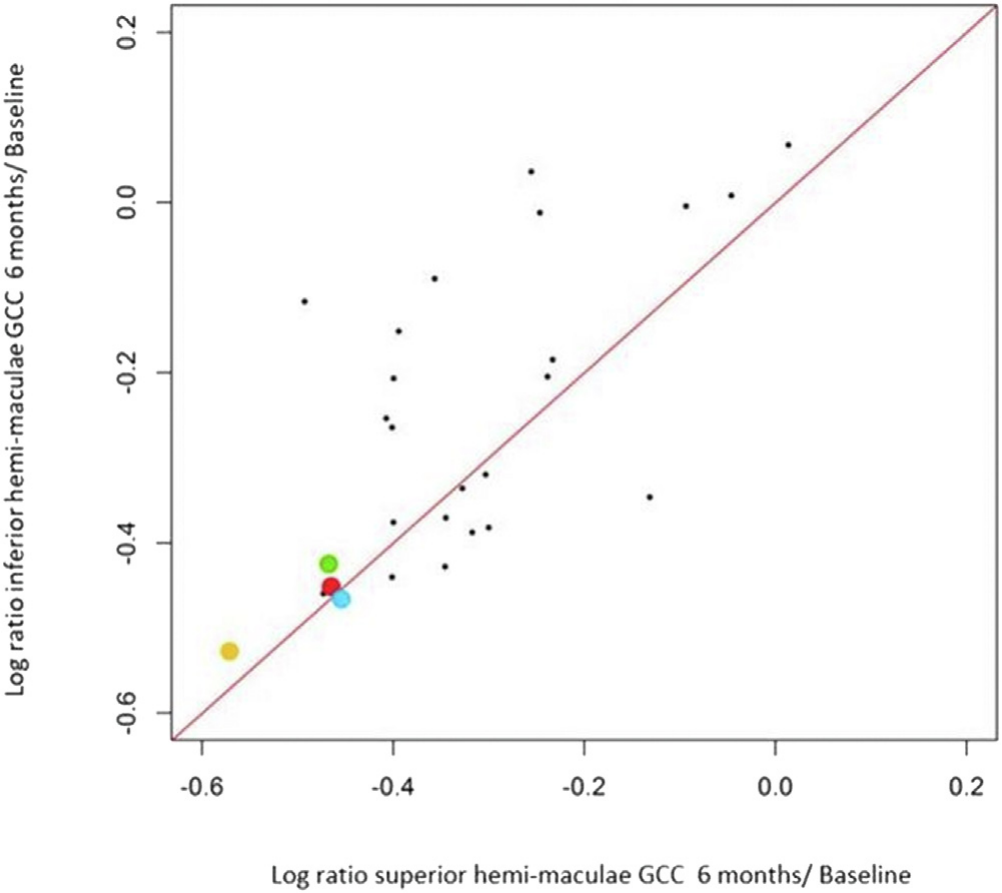
Log ratio of ganglion cell complex (GCC) thickness in the superior and inferior hemimaculae between 6 months and baseline. Patients with microcystic macular edema (MME) had significant thinning of both the superior and inferior hemimaculae (case 1: blue dot, case 2: green dot, and case 3: red dot). One additional patient of the cohort who had significant thinning of both the superior and inferior hemimaculae at 6 months had also significant MME observed (yellow dot).

At baseline, mean INL thickness in these 3 cases was 48.9 ± 17.03 μm in the affected eyes compared with 45.3 ± 1.7 μm in the unaffected eyes (*P* = 0.77). Inner nuclear layer thickening was greatest in case 2 associated with the greatest microcystic change (mean INL thickness: 68.4 and maximal INL thickness 137 μm). At the final follow-up visit (range, 2–3 years postepisode), no significant INL thinning was observed between the affected and unaffected eyes (40.7 ± 5.3 μm and 39.4 ± 2.0 μm, respectively). However, we observed an upward trend in affected eyes, possibly related to the continuing presence of microcystic change. Significant GCC thinning was observed at 6 months in all patients compared with the unaffected eye in the superior hemimaculae (mean loss of GCC thickness, −28.2 ± 5.2 μm [−33.3%, range, −22.3 to 30.3 μm]) and in the inferior hemimaculae (mean loss of GCC thickness, −30.7 ± 5.6 μm [−31.0%, range, −24.3 to 34.8 μm]) (Fig 2). We observed a significant GCC thinning in both hemimaculae in patients with MME compared with the entire cohort, in which greater involvement of the superior hemimacula is shown (mean superior hemimacula thinning: −23.01 ± 9.33, −27.9%, range, 1–40.7 μm, mean inferior hemimacula thinning: −19.14 ± 12.1 μm, −28.51%, range 5 to –39 μm; Fig 2). In 2 patients with similar lesion of GCC, only 1 had also MME (Fig 2). This additional patient of the cohort who had significant thinning of both the superior and inferior hemimaculae at 6 months had also significant MME observed (yellow dot in Fig 2). The other case without MME had a better fovea threshold (25 dB), suggesting a better residual function than the other (mean ± SD: 16 ± 2.6). Finally, in these 3 patients, we observed significant thinning of the RNFL at 6 months (mean pRNFL, 57.7 ± 2.5 μm; temporal RNFL, 48.7 ± 1.5; nasal pRNFL, 56 ± 6.2; inferior pRNFL, 62 ± 2.6 μm; and superior pRNFL, 63.7 ± 3.5). Mean pRNFL was especially thin compared with the overall cohort (mean pRNFL, 62.3 ± 9.7 μm; median, 61 μm; temporal pRNFL, 51.7 ± 15.5 μm; median, 49.5 μm, nasal pRNFL, 60.1 ± 11.8 μm; median: 49.5 μm; inferior pRNFL, 74.9 ± 24.4 μm; median, 66 μm; and superior pRNFL, 62.78 ± 13 μm; median, 62 μm).

### Case Histories

#### Case 1

**Figure 3:**
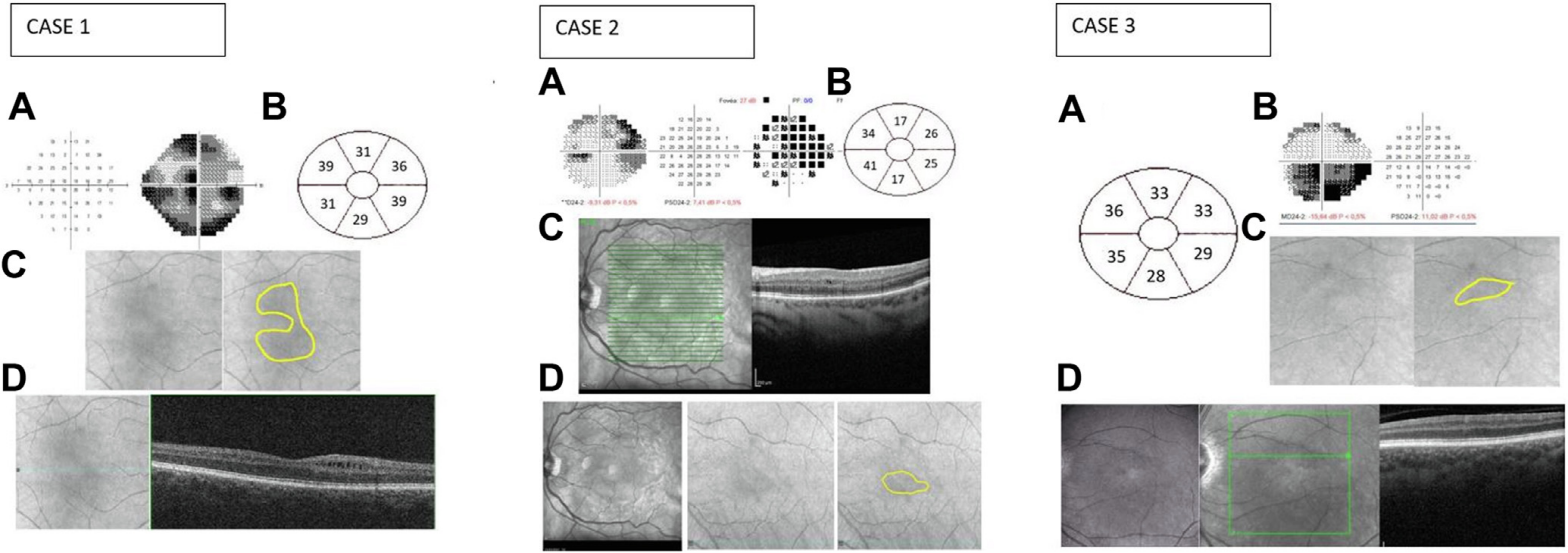
Case 1. **A**, Visual field showing central involvement. **B**, Illustration of Δ ganglion cell layer (GCL) thickness. **C**, Cirrhus high-definition OCT (HD-OCT) fundoscopic images showing a hypointense circular distribution of the inner nuclear layer cystic change. **D**, En face Cirrus OCT showing microcystic macular change (case 1). Case 2. **A**, Visual field showing a superior altitudinal defect. **B**, Illustration of corresponding ΔGCL (μm) thinning between the affected eye at 6 months and the unaffected eye. **C**, En face Heidelberg Retina Angiograph (HRA) OCT (Heidelberg Engineering) showing microcystic macular change located in the inferior hemimacula. **D**, HRA infrared imaging and Cirrhus HD-OCT funduscopic imaging showing an inferior hypointense distribution of the vacuoles. Case 3. **A**, Illustration of corresponding ΔGCL (μm) thinning between the eye before the event and 6 months postepisode. **B**, Visual field showing a superior altitudinal defect. **C**, HD-OCT funduscopy images showing hypointense lesion corresponding to location of microcyst. **D**, HRA en face OCT (Heidelberg Engineering) showing inner nuclear layer microcysts in the inferior hemimacula. This cystic change develops over time, is longstanding, may be permanent, and corresponds with the ganglion cell complex thinning and visual field loss. For each patient, the distribution of cystic change was confirmed by inspection of the horizontal scans.

A 42-year-old man was referred because of sudden painless loss of vision in his right eye, which developed 4 days previously. At presentation, the BCVA was 1/20 in the right eye and 10/10 in the left eye. Funduscopy showed an optic disc swelling and hemorrhages in the right eye. In the left eye, a crowded disc was noted. A diffuse visual field defect with a central involvement was observed in the affected eye (Fig 3). OCT showed significant thickening of the mean pRNFL (363 μm) and a normal thickness of GCC (85 μm). Peripapillary microcystic edema in the INL was also observed on the macular OCT. Fundus fluorescein angiography showed a superior hypoperfusion of the optic disc and a late leakage around it. No choroidal filling defect was observed.

Complete blood count and inflammatory biomarkers (erythrocyte sedimendation rate or C-reactive protein) were within normal limits. However, hypercholesterolemia (low-density lipoprotein [LDL], 121 mg/dL; reference range, < 100 mg/dL) was noted. Severe obstructive sleep apnea was diagnosed following polysomnography, and preexisting hypertension was shown to have been inadequately treated. Magnetic resonance imaging with and without gadolinium enhancement of the brain demonstrated evidence of small vessel disease. A diagnosis of NA-AION was made.

**Figure 4:**
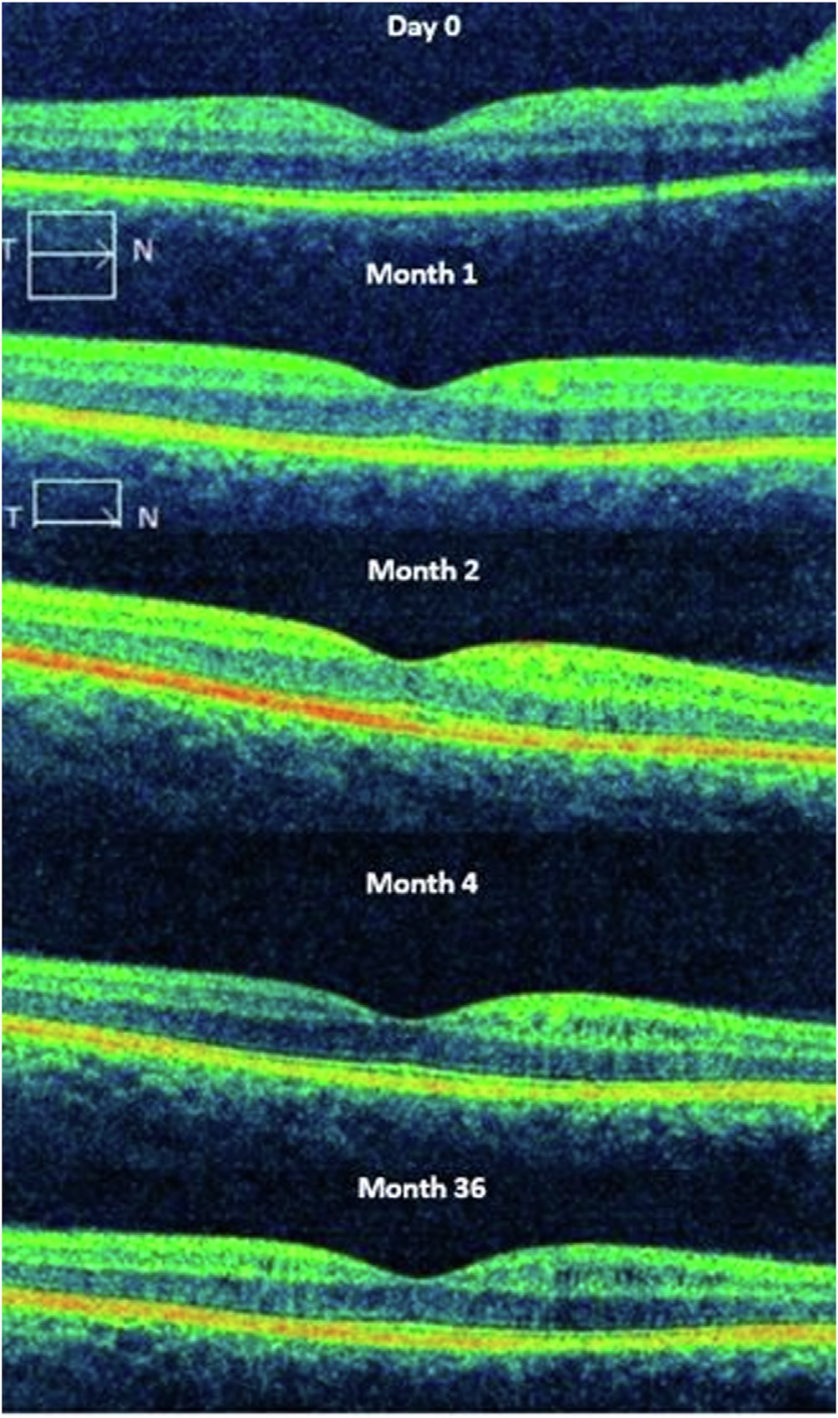
Evolution of the microcysts in the inner nuclear layer that persist over time (case 1) with spectral domian-OCT B-scans (high-definition-OCT Cirrus, Carl Zeiss Meditec).

At follow-up visits, OCT showed a diffuse thinning of the GCC (55 μm at 3 and 6 months) and a significant thinning of the mean pRNFL (64 μm at 3 months and 55 μm at 6 months). Microcysts were observed in the INL in a perifoveal circular distribution. The cysts appeared similar to those seen at presentation apart from the location (Fig 4). The microcysts were first seen 4 months postepisode in the superior hemimacula, corresponding to the greater involvement of the inferior visual field. At 6 months cystic change in the inferior and central regions of the macula was observed and persisted unchanged at the most recent assessment (34 months; Fig 4).

#### Case 2

A 67-year-old man, seen for the first time in 2019, experienced sudden loss of vision associated with a superior visual field defect in his left eye (Fig 3). He had a medical history of treated hypertension and had had a pacemaker implanted. He was borderline overweight (body mass index, 24.3). Polysomnography was within normal limits.

At presentation, BCVA was 10/10 in the right eye and 1.5/10 in the left eye. Examination showed the left optic disc swelling. Perimetry revealed a superior visual field defect with a nasal step and central involvement. Early phases of fundus fluorescein angiography showed a superior sectoral delayed filling of the optic disc, confirming the diagnosis of NA-AION.^23^ Late leakage at the macula was not observed, excluding local vasculopathy. Macular OCT showed subfoveal fluid and microcysts in the INL, which resolved within 1 month as did the pRNFL thickening. Ganglion cell complex analyses were difficult to interpret because of segmentation failure (mean GCC, 70 μm) and pRNFL was thickened (mean pRNFL, 189 μm).

Ophthalmic and neurologic examination were otherwise normal. Complete blood count, blood chemistry, and inflammatory biomarkers were within the respective reference ranges. Magnetic resonance imaging with and without gadolinium enhancement of the brain and orbits was normal.

Six months postepisode, pRNFL was reduced at 58 μm and GCC was thinned (mean GCC, 68 μm). At 1 year follow-up, MME was observed to be limited to the inferior hemimacula (Fig 3) matching the area of greater GCC loss.

#### Case 3

A 52-year-old truck driver presented with blurred vision in his left eye. He had experienced previously blurred vision from an episode of NA-AION in the fellow eye. Best-corrected visual acuity was 1/20 in the right eye and 8/10 in the left eye. A swollen optic disc in the affected eye and diffuse pallor in the fellow eye were observed. The visual field showed an inferior altitudinal defect (Fig 3). Mean pRNFL and GCC were 237 and 67 μm, respectively. Macular OCT scans revealed the traction of the retina induced by the disc swelling associated with fluid between the retinal pigmentary epithelium and the outer nuclear layer together with cystic change in the INL. Fundus fluorescein angiography was not performed at presentation.

Hypercholesterolemia (total cholesterol: 220 mg/dL; reference range, < 190 mg/dL; LDL: 143 mg/dL; reference range < 100 mg/dL) and a slightly elevated glycosylated hemoglobin (43 mmol/mol; reference range, 20–42 mmol/mol) were noted although his complete blood count and inflammatory biomarkers (erythrocyte sedimentation rate or C reactive protein) were within the reference ranges. Polysomnography was performed because of a history of snoring and mild obstructive sleep apnea was diagnosed. Magnetic resonance imaging with and without gadolinium enhancement of the brain was within normal limits.

Thinning of the GCC was observed (55 μm at 3 months and 52 μm at 6 months) and the pRNFL (79 μm at 3 months and 60 μm at 6 months). Microcysts were observed in the superior hemimaculae 6 months postepisode and were persistent at the latest follow-up (24 months postepisode; Fig 4).

## Discussion

A degenerative change in the INL following ganglion cell loss was first reported in 1963^24^ in postmortem studies of primates following chiasmal transection and shown a few years later in postmortem studies of human cases of optic neuropathy.^25^ In the primate study, the cystic degeneration was distributed in an annular perifoveal distribution from approximately 500 μm out, with the foveal margin extending over a further 1 mm, where the ratio of Müller cells to cone bipolar cells is low (1:4 rising to 1:1 at 3 mm eccentricity), although Müller cell to total bipolar cell ratio remains more constant.^26^ Histologic examination in the primate study showed debris and tissue in the larger cysts^24^—confirming that is not edema—and some authors have suggested that degeneration of the Müller cells plays a key role because the MME is localized in the poorly perivascularized rim.^1^^,^^27^^,^^28^ Müller cells are glial cells that provide metabolic and structural support to retinal neurons.^29^ The microcystic appearance observed in the INL may correlate with the vertical orientation of Müller cells.^30^

Following the use of OCT, it has become possible to identify acute and chronic cystic change in the INL in daily practice and the clinical spectrum of appearances designated MME has widened in recent years.^18^ Indeed, it has been described not only in retinal pathology but also in severe optic neuropathy with no known associated retinal pathology and this etiological diversity has led to some confusion among clinicians. There is no consensus regarding the pathogenesis of MME; some authors have suggested that it may be related to retrograde transsynaptic degeneration of bipolar cells^14^^,^^25^ and others have suggested that it may be related to Müller cell dysfunction.^28^^,^^31^

**Figure 5:**
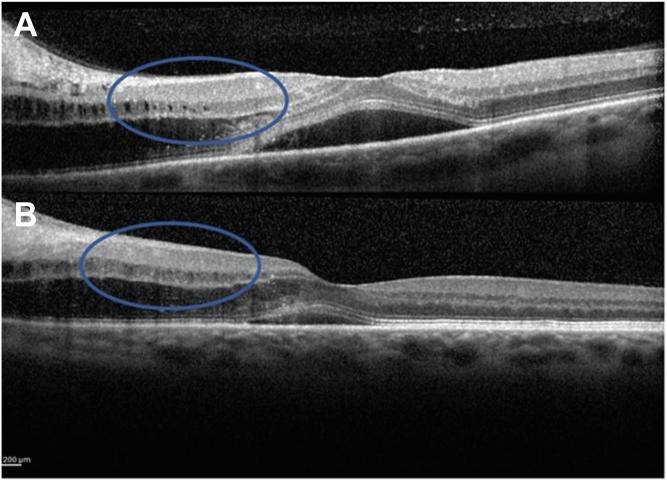
Spectral domain-OCT (Heidelberg Engineering) imaging. Extension of microcystic change in the inner nuclear layer close to the fovea in (**A**) one case from the cohort (cohort case) and (B) case 3. These changes correspond to the degree of optic disc swelling and are reversible with resolution of the retinal edema.

Initially, the 3 patients described above had transient reversible microcystic changes affecting the INL. We propose that this is related to the transudation of fluid from the ischemic optic nerve head sectors extending within and under the retina. This finding recovered spontaneously after 1 month. These microcystic changes were observed in the peripapillary region and were associated with fluid localized between the ellipsoid zone and the outer plexiform layer in the parafoveal region (Fig 1). Because we are describing this microcystic change in the peripapillary region, and as it is likely to be genuine interstitial edema, a better term could be *microcystic peripapillary edema* within the INL although it can extend into the macula (Fig 5). Subsequently, these 3 patients developed the now *classical* MME in the perifoveal region, which we have shown may be very longstanding, perhaps permanent.

Acute microcytic change associated with disc swelling is probably caused by a disruption of the blood–retinal barrier, as proposed by Gelfand et al,^5^ leading to an overwhelming of Müller cell function. Over the past 5 years, various authors have described the presence of a paravascular transport system in the retina and around the optic nerve similar to the glymphatic system existing in the brain.^31^, ^32^, ^33^ In a human postmortem study assessing a cross-section of the optic nerve, Wostyn et al^33^ found an accumulation of India ink in paravascular spaces around the central retina vein and artery, which could correspond to an optic nerve glymphatic system. It has also been suggested that a hyporeflective area around the vessel walls observed on OCT provide evidence for such a system.^32^ In NA-AION, the glymphatic system could be compromised along with the venous drainage by some common mechanisms observed in ischemia of the optic nerve. Müller cells and the superficial and deep vascular plexuses under normal conditions play a role in the reabsorption of fluid^28^^,^^31^; however, following arterial hypoperfusion and extensive extravasation of fluid from capillaries and venules, Müller cells and other mechanisms of tissue homeostasis are saturated. This explains why we observe the OCT microcysts in the INL, similar to what has been observed in MME,^1^ although the retinal distribution differs. Another factor may be that the transudate forming subretinal fluid might induce traction on the Müller footplates, explaining the cystic appearance observed.^7^^,^^15^ Once the disc swelling had resolved, we observed a complete resolution of the cystic change.

In contrast, MME appeared a few months post episode—after 4 months in the first case—and persisted over time (at least 34 months). Burggraaff et al^18^ have described that MME was transient in 84% of their series, but most of the cases were age-related macular degeneration and epiretinal membrane traction, in which the pathogenesis may be quite different. In our cases, we observed the persistence of MME, indicating a likely permanent change as also observed by Abegg et al.^14^ Therefore, retinal ganglion cell loss inducing vitreoretinal traction on the Müller cells is a less likely pathogenesis than a metabolic change related to retrograde transsynaptic degeneration,^8^^,^^16^ either dysfunction of existing Müller cells^3^ or empty space following Müller cell degeneration that filled with fluid.^13^ Indeed, the stationary nature of the cysts over a long follow-up period (3 years), suggesting a fixed change is very unlikely to be interstitial edema (Fig 4).

In NA-AION, patients present with an altitudinal defect corresponding to the loss of ganglion cells induced by hypoperfusion (infarction) of ≥ 1 optic disc sectors. We observed a strong relationship between the location of MME and the thinning of ganglion cells, corresponding to the visual field defect. Indeed, MME seems to respect the horizontal meridian in cases 2 and 3, compatible with an effect of retrograde degeneration. Moreover, greater RNFL and GCC thinning were observed in these patients compared with the entire cohort (Fig 2). Contrary to the conclusions of Lee DH et al,^17^ in NA-AION, a poorer overall visual outcome is not necessary to observe MME, but rather a significant localized loss of retinal ganglion cells is key. Microcystic macular edema was indeed observed by the authors (A.C.C., G.T.P.) in 2 patients who had poor vision (cases 1 and 2) but also in a patient with good overall vision because central vision was spared (case 3). The question arises as to the impact of the INL change on visual function. Clearly, it signifies greater retinal ganglion cell loss but whether any additional impairment of function occurs is unknown. To investigate this question in NA-AION would require detailed visual function analysis and possibly electroretinography concentrating on the macula, comparing cases with comparable visual field loss with and without MME.

In conclusion, our study indicates 2 causes of MME in the same patients suffering from NA-AION, 1 reversible and the other likely permanent. Müller cell dysfunction seems to play a key role in this finding and offers a potential explanation for both conditions. This finding highlights the distinction between genuine edema related to transudation of fluid (here associated with optic disc swelling) and the phenomenon observed in RM related to the degree of RNFL loss. We consider that MME might be considered a misnomer if there is no tissue edema and, until the exact pathology is identified, would recommend the use of the term *RM*.^13^^,^^16^ The distribution of the change corresponded in our cases to both the more significant visual field loss and the greater GCC thinning. It appeared after a time interval of some weeks and may persist for many months or years. These are not the characteristics that would be expected if tissue edema were the underlying mechanism giving rise to the INL cystic change. Nonarteritic anterior ischemic optic neuropathy is also a useful model because of the localized retinal change corresponding to localized axonal loss, as has also been found in the nasal hemimacula in chiasmal compression.^34^
